# Corrosion Damage and Life Prediction of Concrete Structure in a 41-Year-Old Steelworks

**DOI:** 10.3390/ma15175893

**Published:** 2022-08-26

**Authors:** Yao Lv, Ditao Niu, Xiguang Liu, Yue-Chen Li

**Affiliations:** 1 School of Civil Engineering, Xi’an University of Architecture and Technology, No. 13 Yanta Rd., Xi’an 710055, China; 2 State Key Laboratory of Green Building in Western China, Xi’an University of Architecture and Technology, No. 13 Yanta Rd., Xi’an 710055, China

**Keywords:** environmental characteristic, concrete, neutralization depth, compressive strength, life prediction

## Abstract

Iron and steel industry emits a large amount of CO_2_ and SO_2_ in the process of steelmaking, and these acid gases lead to the serious corrosion damage of concrete structures. In this paper, the environmental characteristics and corrosion degree of concrete in a 41-year-old steelworks were investigated, and the neutralization life prediction of the concrete structure was carried out. The results showed that the temperature, relative humidity, CO_2_ concentration, and SO_2_ concentration in the steelworks were 1.32, 0.62, 1.28, and 13.93 times higher than those of the general atmospheric environment, respectively. These environmental characteristics in various sections were significantly different. The appearance change of concrete in the ingot casting bay was more serious than that of concrete in the billet bay. Both the compressive strength of concrete in the ingot casting bay and billet bay decreased, and the strength in the billet bay was relatively low. The neutralization depth of concrete in the ingot casting bay was 2.35 times larger than that of concrete in the billet bay. The prediction model of concrete neutralization depth was established, and the remaining neutralization service life in the ingot casting bay and billet bay were 194.68 a and 202.07 a, respectively.

## 1. Introduction

Steel is the most widely used and consumed alloy in the world. China is the largest steel producer in the world [1]. The steel output of China was 996 million tons in 2019, which accounted for 53.3% of the global steel output [2]. The main energy source of China’s iron and steel sector is coal [3], and CO_2_ and SO_2_ are emitted in the process of combustion. Moreover, iron ore contains the sulfur element, which inevitably produces SO_2_ in the process of mining and processing [4]. According to statistics, China’s total CO_2_ emissions in 2019 were 11.198 billion tons [5], and the CO_2_ emissions that generated by China’s steel industry accounted for 14% of total CO_2_ emissions [6]. The SO_2_ emissions generated by China’s steel industry in 2015 were 1.37 million tons, which accounted for 7.36% of China’s total SO_2_ emissions [7].

The pore-solution pH of ordinary Portland cement concrete is usually between 12.5 to 13.5 [8], and this high alkalinity environment prevents the protective film of iron oxides around rebars from destroying [9]. CO_2_ and SO_2_ can react with alkaline hydration products. Thrangavel [10] found that CO_2_ reacted with Ca(OH)_2_ and decreased the pore-solution pH of concrete, and Niu [11] and Pavlik [12] found that SO_2_ could also reduce the alkalinity of concrete. Once pore-solution pH drops to about 9, the protective film of the rebars is destroyed, and the rebars begin to corrode [13]. The corrosion products accumulate in the pore structure and generate expansion pressure on the surrounding concrete [14]. Excessive pressure leads to the initiation and propagation of cracks in concrete, thereby reducing the load carrying capacity of concrete structures. Therefore, it is necessary to develop strategies for the CO_2_ and SO_2_ resistance of concrete. To achieve this goal, the study on the neutralization of concrete under the combined action of CO_2_ and SO_2_ should be accomplished.

At present, the research on concrete neutralization mainly focuses on the action of single CO_2_ or SO_2_. According to the theoretical analysis [15], laboratory accelerated test [16,17], and field investigation [18,19] of concrete carbonation, CO_2_ reacted with calcium compounds of hydration products to form CaCO_3_. SO_2_ reacted with all calcium compounds, including CaCO_3_, and converted them into calcium sulfates and calcium sulfoaluminates [20]. Calcium sulfoaluminates were stable in a certain pH range [21]. Once pH fell to a certain value, calcium sulfoaluminates decomposed. It was reported that the decomposition of ettringite and monosulfoaluminate hydrate at 20 °C were at pH ≤ 10.7 and pH ≤ 11.6, respectively [22]. The decomposition products were gypsum, alumina gel, and water [23]. CaCO_3_ increased the compactness of concrete, so the carbonation of concrete increased the compressive strength. Li [24] and Rostami [25] verified this conclusion through a carbonation test. Calcium sulfates and calcium sulfoaluminates greatly increased the solid volume of concrete, and the mass and compressive strength increased at first and then decreased [26].

There is little research on concrete neutralization under the combined action of CO_2_ and SO_2_, mainly focusing on the neutralization mechanism. Pavlik [12] investigated the degradation of concrete by flue gases in a power plant. The results showed that the corroded concrete was divided into a soft disintegrated zone, sulfated zone and carbonated zone. Scholl [27] found that the corrosion products of concrete were calcite, gypsum, and ettringite under the combined action of SO_2_ and CO_2_. Lean [28] observed that the diffusion rate of CO_2_ in concrete was faster than that of SO_2_ at the same volume concentration. Furthermore, the concentration of CO_2_ was higher than that of SO_2_ in an industrial environment. Therefore, the essence of concrete sulfuration was the reaction between SO_2_ and carbonation products under the action of CO_2_ and SO_2_.

To sum up, there are few research results on the concrete neutralization under the combined action of CO_2_ and SO_2_, especially in terms of the degree of concrete performance degradation. Therefore, the corrosion damage and life prediction of concrete structures in a 41-year-old steelworks of Wuhan Iron and Steel Corp. (WISCO) were studied in the present work. The environmental characteristics such as temperature, relative humidity, CO_2_ concentration, and SO_2_ concentration in various sections of the steelworks were monitored, respectively. The appearance, cover thickness, neutralization depth, and compressive strength of concrete in the ingot casting bay and billet bay were analyzed, respectively. The prediction model of neutralization depth of concrete in the steelworks was established, and the remaining neutralization service life in the ingot casting bay and billet bay were predicted.

## 2. Materials and Methods

The industrial environment of steelworks is a typical environment with high temperatures and high concentrations of acid gas. The temperatures of molten iron and molten steels are 1250–1350 °C and 1600–1700 °C, respectively. Therefore, steelworks are exposed to a high-temperature environment for a long time. Besides, a large amount of acid gases, such as CO_2_ and SO_2_, are generated in the process of steelmaking. Therefore, the corrosion damage to concrete structures in the steelworks is serious. Taking a 41-year-old steelworks of WISCO as an example, the research group investigated the environmental characteristics and corrosion damage of concrete structures from August to October 2013.

### 2.1. Project Brief

WISCO was located in Qingshan District, Wuhan City. The main building of the steelworks was built in 1972, and it was divided into seven sections: charging bay, acceptance bay, converter bay, casting bay, ingot casting bay, billet bay, and granulating slag bay. Among them, the ingot casting bay and billet bay were the concrete bent construction. The ingot casting bay was a single slope bent structure with a span of 24 m and a column spacing of 37. The bent frame column of the billet bay adopted the diagonal web member double-wing column.

### 2.2. Materials

The concrete consisted of ordinary Portland cement, with natural river sand as the fine aggregate and crushed limestone as the coarse aggregate. Both the design thicknesses of concrete cover in the ingot casting bay and billet bay were 30 mm, and the concrete mark of the column was 400. According to Chinese standard GB 50010-2010 [29], the characteristic value of concrete compressive strength was 38 MPa.

### 2.3. Experimental Methods

#### 2.3.1. Temperature Monitoring

The temperature in the steelworks was tested by the temperature and humidity recorder (8829, AZ Instrument, Taichung, China), and the data were collected hourly. The layout of measuring points was based on the Chinese standard GB/T 18204.13-2000 [30]. There were five measuring points in each section of the steelworks, and they were arranged on two diagonals with the shape of plum blossom. The height of measuring points was 0.8–1.6 m from the ground, and the distance between the measuring points and the heat source or wall was not less than 0.5 m. The average of all measuring points in each section was the average temperature in the section. The average of all sections was the average temperature in the steelworks.

#### 2.3.2. Relative Humidity Monitoring

The relative humidity in the steelworks was tested by the temperature and humidity recorder, and the data were collected hourly. The arrangement of measuring points was the same as that of measuring points for temperature monitoring. The average of all measuring points in each section was the average relative humidity in the section. The average of all sections was the average relative humidity in the steelworks.

#### 2.3.3. CO_2_ Concentration Monitoring

The CO_2_ concentration in the steelworks was monitored by using the carbon dioxide detector (7752, AZ Instrument, Taichung, China), and the data were collected hourly. The layout of measuring points was the same as that of measuring points for temperature monitoring. The average of all measuring points in each section was the average CO_2_ concentration in the section, and the average of all sections was the average CO_2_ concentration in the steelworks.

#### 2.3.4. SO_2_ Concentration Monitoring

The SO_2_ concentration in the steelworks was monitored by using the sulfur dioxide detector (HD5, Huideng, Nanjing, China), and the data were collected hourly. The layout of measuring points was the same as that of measuring points for temperature monitoring. The average of all measuring points in each section was the average SO_2_ concentration in the section, and the average of all sections was the average SO_2_ concentration in the steelworks.

#### 2.3.5. Thickness of Concrete Cover

The thickness of concrete cover in the steelworks was tested by a rebar scanner (PROFOMETER-5, Proceq, Schweitzenbach, Switzerland) with a measuring range of 70 mm. Concrete columns were randomly selected to test the thickness of concrete cover. Each column had 30 testing zones, which were evenly distributed on two symmetrical measurable surfaces of the column. The thickness of concrete cover in each testing zone was tested along the direction of the main reinforcement of the column, and the average value of all testing zones in each column was the average thickness of concrete cover of the column.

#### 2.3.6. Neutralization Depth of Concrete

The neutralization depth test method of concrete in Chinese standard JCJ/T 23-2011 [31] was adopted in this experiment. The selection of the concrete columns was the same as that of the columns for testing the thickness of concrete cover. There were three testing zones in each column. Take a hole with a diameter of 15 mm in each testing zone, and the depth of the hole should be larger than its neutralization depth. Measure the neutralization depth with a 1–2% phenolphthalein alcohol solution (alcohol solution contains 20% distilled water). The test was carried out by using a carbonation depth meter (HC-TH01, Hichance, Beijing, China). Each hole was measured three times, and the average of three measurements was considered the neutralization depth of the testing zone. The average of all the testing zones in each column was the neutralization depth of the concrete column.

#### 2.3.7. Compressive Strength of Concrete

The compressive strength of concrete was tested by the rebound method according to JCJ/T 23-2011, and the test was carried out by using a rebound hammer (HT225A, Hichance, Beijing, China). The selection of concrete columns was the same as that of columns for testing the concrete cover thickness. Two testing zones were selected for each column, and they were arranged on two symmetrical measurable surfaces of the column. The measuring points in each testing zone were not less than 16. The measuring points should be evenly distributed, and the distance between two adjacent measuring points should not be less than 20 mm. The distance between the measuring points and the exposed steel bars or embedded parts was not less than 30 mm. The measuring points should not appear on stones and pores, and each measuring point bounced only once. After removing three maximum values and three minimum values, the arithmetic mean value of the remaining measuring points was the average rebound value of the testing zone. The average rebound value of the two testing zones was the final strength of the column. The equivalent value of concrete compressive strength was converted according to the rebound value of compressive strength and the neutralization depth.

## 3. Results and Discussion

### 3.1. Survey of Environmental Characteristics

#### 3.1.1. Temperature

**Figure 1:**
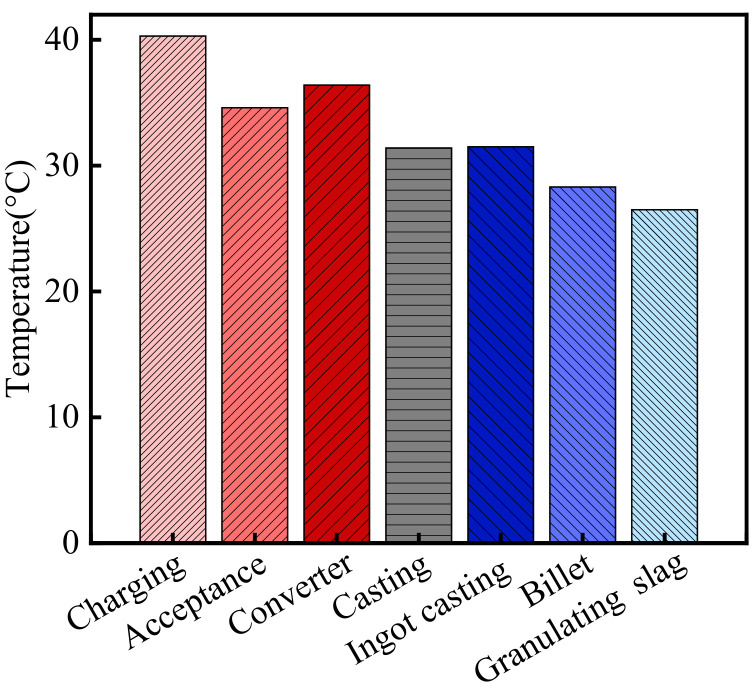
The temperature in the steelworks.

The temperature in the steelworks is shown in Figure 1. As shown in the figure, the average temperature in the steelworks was 32.7 °C from August to October 2013. According to the meteorological data, the atmospheric temperature in Wuhan was 24.8 °C during the same period [32]. Therefore, the temperature in the steelworks was 1.32 times higher than that of the general atmospheric environment. Moreover, there were significant differences in the temperatures in various sections. The temperatures in the process sections in front of and in the middle of the furnace, such as the charging bay, acceptance bay, converter bay, casting bay, and ingot casting bay, etc., were higher than those in the post furnace process sections, such as billet bay and granulating slag bay. The temperatures in the charging bay, acceptance bay, converter bay, casting bay, ingot casting bay, billet bay, and granulating slag bay were 40.3, 34.6, 36.4, 31.4, 31.5, 28.3, and 26.5 °C, respectively. The temperature in the charging bay was the highest, and it was 1.63 times higher than that of the general atmospheric environment.

#### 3.1.2. Relative Humidity

**Figure 2:**
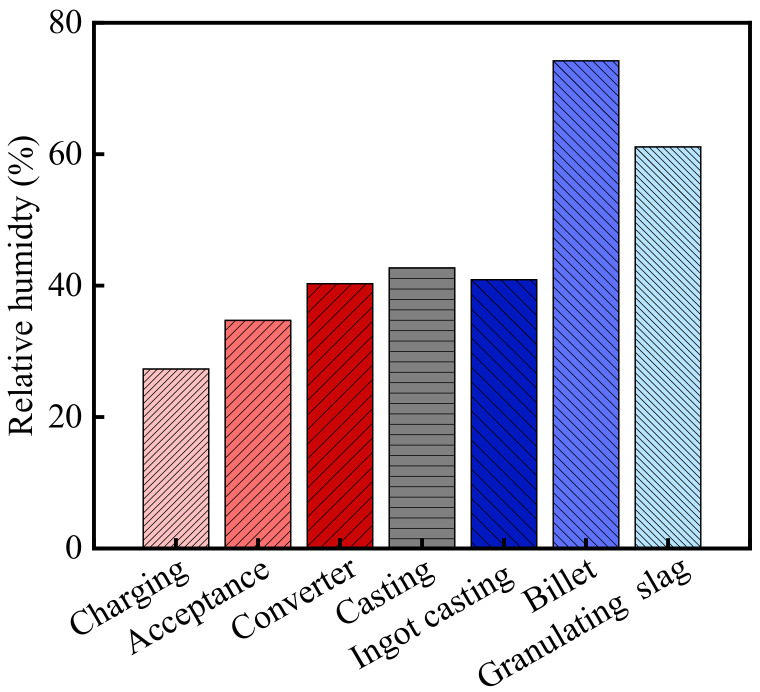
Relative humidity in the steelworks.

The relative humidity in the steelworks is shown in Figure 2. As shown in the figure, the average relative humidity in the steelworks was 45.9% from August to October 2013. According to the meteorological data, the relative humidity of the atmospheric environment in Wuhan was 73.7% during the same period [32]. Therefore, the relative humidity of the general atmospheric environment was 1.61 times higher than that in the steelworks. Besides, the relative humidity in various sections was significantly different, and there was a thermal hydraulic coupled process between relative humidity and temperature. As the temperature increased, the relative humidity showed a downward trend. The relative humidity in the post furnace process sections (billet bay and granulating slag bay) was closed to that of the general atmospheric environment, and the humidity in other sections was much lower than that of the general atmospheric environment. The relative humidity in the billet bay and granulating slag bay were 74.2 and 61.1%, respectively. The relative humidity in the charging bay, acceptance bay, converter bay, casting bay, and ingot casting bay were lower, and they were 27.7, 34.7, 40.3, 42.7, and 40.9%, respectively. The relative humidity in the charging bay was the lowest, and it was 0.38 times higher than that of the general atmospheric environment.

#### 3.1.3. CO_2_ Concentration

**Figure 3:**
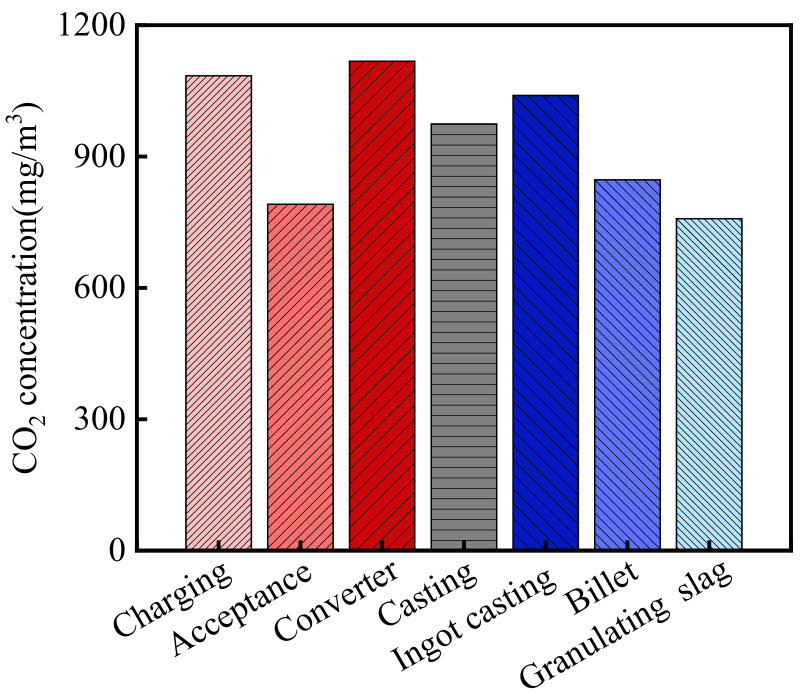
CO_2_ concentration in the steelworks.

The CO_2_ concentration in the steelworks is shown in Figure 3. As shown in the figure, the average CO_2_ concentration in the steelworks was 944.5 mg/m^3^. According to the relevant information from the Wuhan Environmental Protection Bureau (WHEPB), the CO_2_ concentration of the atmospheric environment in the Qingshan District was 740.5 mg/m^3^ [32]. Therefore, the CO_2_ concentration in the steelworks was 1.28 times higher than that of the general atmospheric environment. In addition, there were significant differences in the CO_2_ concentrations in various sections, and the CO_2_ concentrations in the charging bay, acceptance bay, converter bay, casting bay, ingot casting bay, billet bay, and granulating slag bay were 1084.3, 791.2, 1117.6, 974.3, 1039.4, 846.6, and 758.2 mg/m^3^, respectively. Therefore, CO_2_ concentration in all sections of the steelworks was higher than that of the general atmospheric environment, and the maximum value was 1.51 times higher than that of the general atmospheric environment.

#### 3.1.4. SO_2_ Concentration

**Figure 4:**
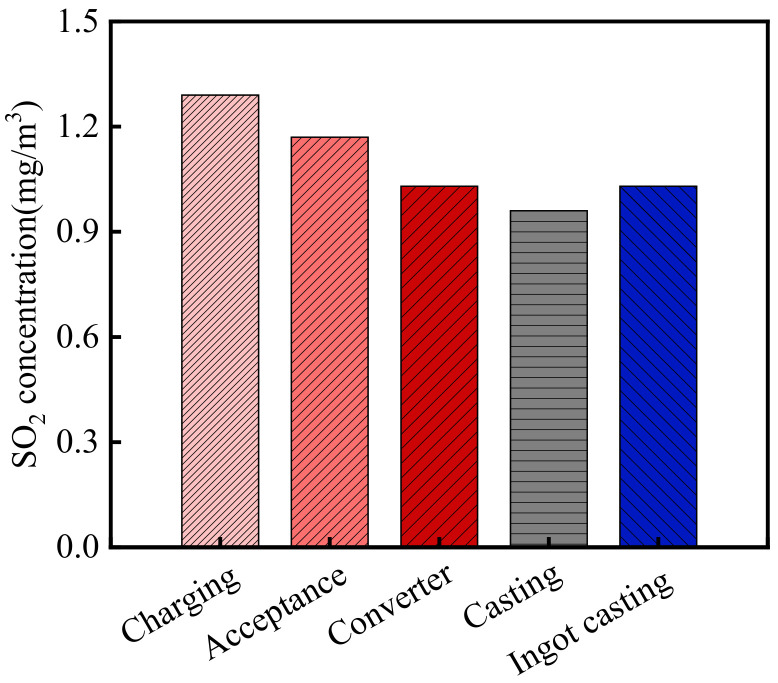
SO_2_ concentration in the steelworks.

The SO_2_ concentration in the steelworks is shown in Figure 4. As shown in the figure, the average SO_2_ concentration in the steelworks was 0.78 mg/m^3^. According to the relevant information from WHEPB, the SO_2_ concentration of the atmospheric environment in the Qingshan District was 0.056 mg/m^3^ [32]. Therefore, SO_2_ concentration in the steelworks was 13.93 times higher than that of the general atmospheric environment. There were significant differences in the SO_2_ concentrations in various sections. The SO_2_ concentrations in the charging bay, acceptance bay, converter bay, casting bay, and ingot casting bay were 1.29, 1.17, 1.03, 0.96 and 1.03 mg/m^3^, respectively. No obvious SO_2_ was detected in the billet bay and granulating slag bay. Therefore, the SO_2_ concentration in the charging bay was the largest, which was 20.04 times higher than that of the general atmospheric environment.

### 3.2. Analysis of Corrosion Degree of Concrete Structure

#### 3.2.1. Appearance of Concrete

**Figure 5:**
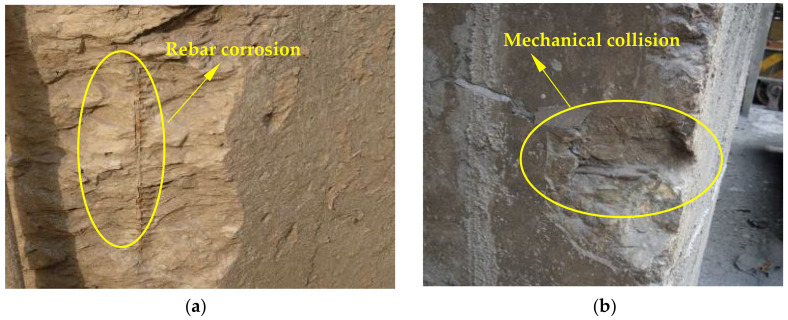
The appearance of concrete structures in the steelworks: (**a**) Ingot casting bay; (**b**) Billet bay.

The appearance of concrete in the ingot casting bay and billet bay of the steelworks is shown in Figure 5. As shown in the figure, although the concrete columns in the ingot casting bay were protected by external bricks, some rebars were still exposed and rusted (Figure 5a). There was some corner damage to the concrete column in the billet bay caused by mechanical collision (Figure 5b). SO_2_ existed in the ingot casting bay and reacted with hydration products of concrete, resulting in the sulfuration reaction of concrete. Sulfuration could lead to the spalling and damage of concrete [11]. Therefore, the concrete cover fell off, and the rebars were exposed and corroded. However, there was no SO_2_ in the billet bay, and therefore, there was only some damage on the surface of concrete caused by mechanical collision.

#### 3.2.2. Thickness of Concrete Cover

**Figure 6:**
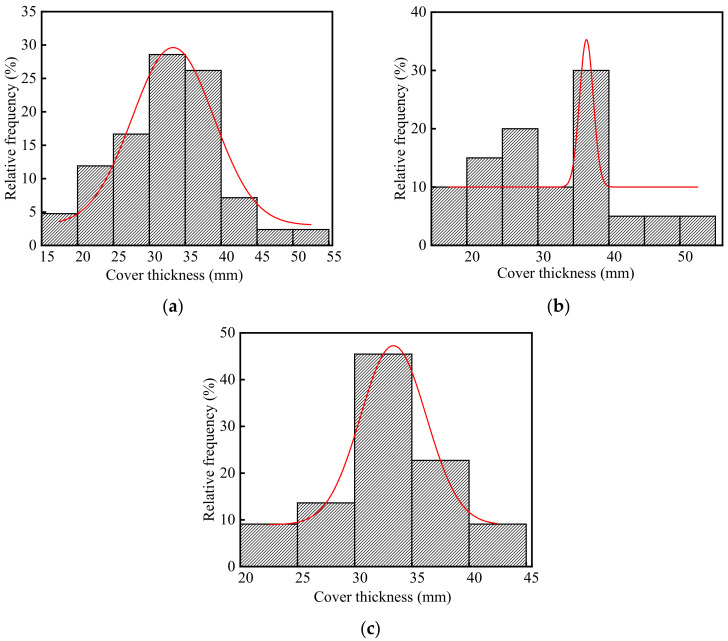
Frequency distribution histograms of concrete cover thickness in the steelworks: (**a**) Both in the ingot casting bay and billet bay; (**b**) Ingot casting bay; (**c**) Billet bay.

The thickness of concrete cover in the steelworks is listed in Table S1 (See Supplementary Materials), and the frequency distribution histograms of concrete cover thickness are shown in Figure 6. As shown in Figure 6a, the average thickness of the concrete cover in the steelworks was 32.42 mm, and the ratio of the test value to the design value was 1.08. This indicated that there was little difference between the test value and the design value of the concrete cover thickness in the steelworks. The coefficient of variation of concrete cover thickness was 0.22, indicating that the discreteness of cover thickness was good. It was found that the *p*-value was 0.81 at a significant level α = 0.05 by using Shapiro-Wilk. Therefore, the thickness of the concrete cover was in accordance with the normal distribution. In summary, the thickness of concrete cover in the steelworks basically met the structural design requirements.

As shown in Figure 6b,c, the average values of concrete cover thickness in the ingot casting bay and billet bay were 32.33 and 32.50 mm, and the coefficients of variation were 0.28 and 0.15, respectively. The results showed that there was little difference between the test value and the design value of the concrete cover thickness in the ingot casting bay and billet bay. By using Shapiro-Wilk, the p values of the concrete cover thickness in the ingot casting bay and billet bay at the significant level α = 0.05 were 0.63 and 0.89, respectively. Therefore, both the thickness of concrete cover in the ingot casting bay and billet bay followed the normal distribution, and they basically met the structural design requirements.

#### 3.2.3. Neutralization Depth of Concrete

**Figure 7:**
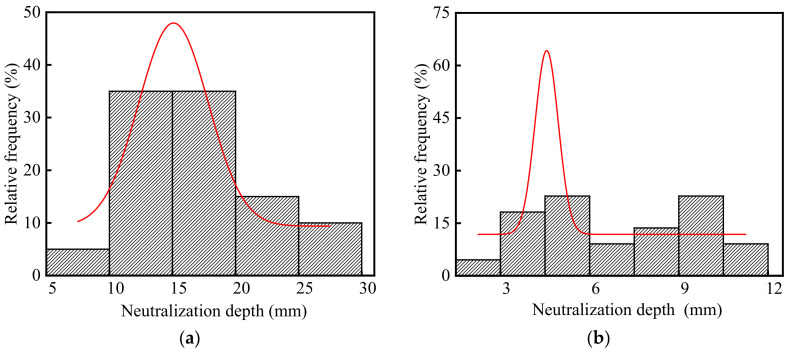
Frequency distribution histograms of concrete neutralization depth in the steelworks: (**a**) Ingot casting bay; (**b**) Billet bay.

The neutralization depth of concrete in the steelworks is listed in Table S1, and the frequency distribution histograms of concrete neutralization depth are shown in Figure 7. The average and standard deviation of concrete neutralization depth in the steelworks were 11.40 and 6.20 mm, respectively. The coefficient of variation was 0.54, and this indicated that the neutralization depth of concrete in the steelworks had a large discreteness. This was because environmental characteristic parameters, especially acid gas concentrations, affected the neutralization depth of concrete. Therefore, the neutralization depth of concrete in different sections varied greatly.

As shown in Figure 7a,b, the neutralization depths in the ingot casting bay and billet bay were different, and the average values were 16.32 and 6.93 mm, respectively. This was because the neutralization depth of concrete increased with the increase in the temperatures [33] and acid gas concentrations [34]. The CO_2_ concentration and temperature in the ingot casting bay were 1.23 times and 1.11 times higher than those in the billet bay, respectively. In addition, the presence of SO_2_ in the ingot casting bay led to the cracking of concrete and accelerated the neutralization rate. Therefore, the neutralization depth of concrete in the ingot casting bay was larger, and it was 2.35 times larger than that in the billet bay.

As shown in Figure 7, the p values of neutralization depth in the ingot casting bay and billet bay were 0.30 and 0.13 at the significant level α = 0.05 by using Shapiro-Wilk, respectively. Therefore, the neutralization depth of concrete followed the normal distribution. The coefficients of variation of neutralization depth in the ingot casting bay and billet bay were 0.31 and 0.41, respectively. Therefore, the neutralization depth in the billet bay had a larger discreteness. This was because that one side of the concrete double leg column in the billet bay was in the indoor environment, and the other side was in the outdoor environment with the influence of climate changes, such as rain, temperature changes, freeze-thaw cycles, etc. The neutralization depths of the indoor concrete columns were less than 8 mm, and those of the outdoor concrete columns were more than 10 mm. Therefore, the discreteness of concrete neutralization depth in the billet bay was relatively large.

#### 3.2.4. Compressive Strength of Concrete

**Figure 8:**
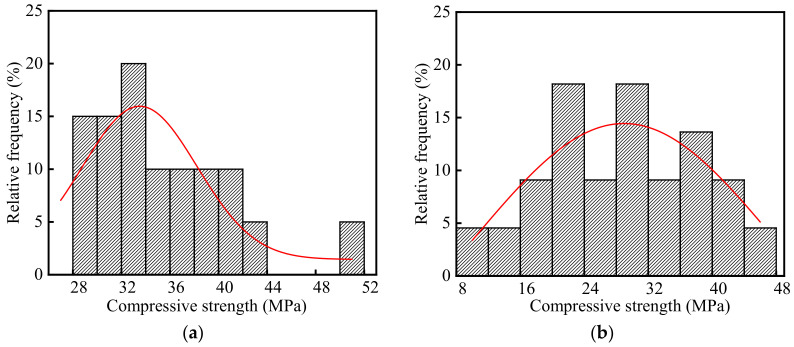
Frequency distribution histograms of concrete compressive strength in the steelworks: (**a**) Ingot casting bay; (**b**) Billet bay.

The compressive strength of concrete in the steelworks is listed in Table S1, and the frequency distribution histograms of concrete compressive strength are shown in Figure 8. As shown in the figure, the average, standard deviation, and variation of coefficient of the compressive strength were 31.96, 8.39, and 0.26 MPa, respectively. The test value of compressive strength was lower than the design value. On the one hand, the creep stress of concrete structures reduced the compressive strength. On the other hand, the decreasing rate of the strength was accelerated by the influence of SO_2_ [26] and some environmental changes [35,36], such as temperature changes, rain, and freeze-thaw cycles, etc.

As shown in Figure 8a,b, the average compressive strength in the ingot casting bay was 1.23 times larger than that of compressive strength in the billet bay. Half of the concrete columns in the billet bay were in the outdoor environment, and therefore, the compressive strength in the billet bay decreased more due to the influence of temperature changes, rain, and freeze-thaw cycles.

As shown in Figure 8, the p values of compressive strength in the ingot casting bay and billet bay were 0.17 and 0.78 at the significant level α = 0.05 by using Shapiro-Wilk, respectively. Therefore, the compressive strength of concrete followed the normal distribution. The coefficients of variation of concrete compressive strength in the ingot casting bay and billet bay were 0.16 and 0.32, respectively. The compressive strength in the billet bay had a larger discreteness. This was because that one side of the concrete columns in the billet bay was in the indoor environment, and the other side was in the outdoor environment affected by climate changes. The compressive strength of the indoor concrete was significantly larger than that of the outdoor concrete.

## 4. Life Prediction of Concrete Structure

### 4.1. Prediction Model of Neutralization Depth of Concrete

It is verified by a large number of researchers that the carbonation depth of concrete varies linearly with the square root of exposure time [37,38,39,40,41,42,43]. The expression used is given in Equation (1).

(1) $$X_{C}=k\sqrt{t}$$

where *X*_C_ is the carbonation depth of concrete, mm; *k* is the carbonation coefficient, mm/d^0.5^; *t* is the carbonation exposure duration, d.

There is only CO_2_ acid gas in the billet bay, and therefore, the neutralization depth of concrete in the billet bay is the carbonation depth. The carbonation depth of concrete is affected by environmental parameters and material properties. In this paper, temperature, relative humidity, and CO_2_ concentration are the environmental parameters, and compressive strength is the material property of concrete. Therefore, Equation (1) can be expressed as:

(2) $$X_{C}=k_{0}k_{1}k_{2}k_{3}\sqrt{t}$$

where *k*_0_ is the undetermined parameter; *k*_1_, *k*_2_ and *k*_3_ are the carbonation influence coefficients of the temperature and humidity, CO_2_ concentration and compressive strength, respectively.

According to the literature [43], the carbonation influence coefficients of temperature and humidity, CO_2_ concentration, and compressive strength can be calculated as follows:

(3) $$k_{1}=2.56\sqrt[{4}]{T}RH\left(1-RH\right)$$

(4) $$k_{2}=\sqrt{\frac{C_{C}}{0.03}}$$

(5) $$k_{3}=\frac{57.94}{f_{\mathrm{cuk}}}-0.76$$

where *T* is the temperature, °C; *RH* is the relative humidity, %; *C*_C_ is the concentration of CO_2_, %; and *f*_cuk_ is the compressive strength of concrete, MPa.

Substituting Equations (3), (4), and (5) into Equation (2), the carbonation depth of concrete can be expressed as:

(6) $$X_{C}=k_{0}\sqrt[{4}]{T}\left(1-RH\right)RH\sqrt{C_{C}}\left(\frac{57.94}{f_{\mathrm{cuk}}}-0.76\right)\sqrt{t}$$

**Figure 9:**
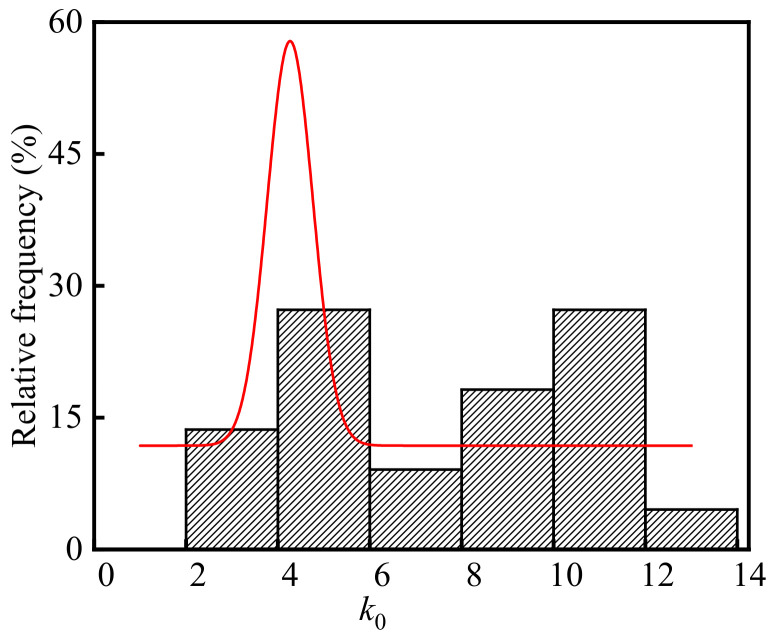
Frequency distribution histogram of *k*_0_.

The neutralization depth of concrete in the billet bay is the carbonation depth. Substituting the test values of the temperature, relative humidity, CO_2_ concentration, concrete compressive strength, concrete neutralization depths, and neutralization ages in the billet bay into Equation (6), *k*_0_ is calculated and the frequency distribution histogram of *k*_0_ is shown in Figure 9. As shown in the figure, the average, standard deviation, and coefficient of variation of *k*_0_ were 7.66, 3.16, and 0.41, respectively. The *p*-value of *k*_0_ was 0.13 at the significant level α = 0.05 by using Shapiro-Wilk. Therefore, *k*_0_ in the steelworks followed the normal distribution.

SO_2_ concentration in the steelworks is much lower than that of CO_2_. However, SO_2_ can lead to the expansion and cracking of concrete, which accelerates the concrete neutralization. Therefore, the neutralization depth of concrete in the ingot casting bay can be expressed as:

(7) $$X=k_{5}X_{C}$$

where *k*_5_ is the neutralization influence coefficient of SO_2_ concentration.

**Figure 10:**
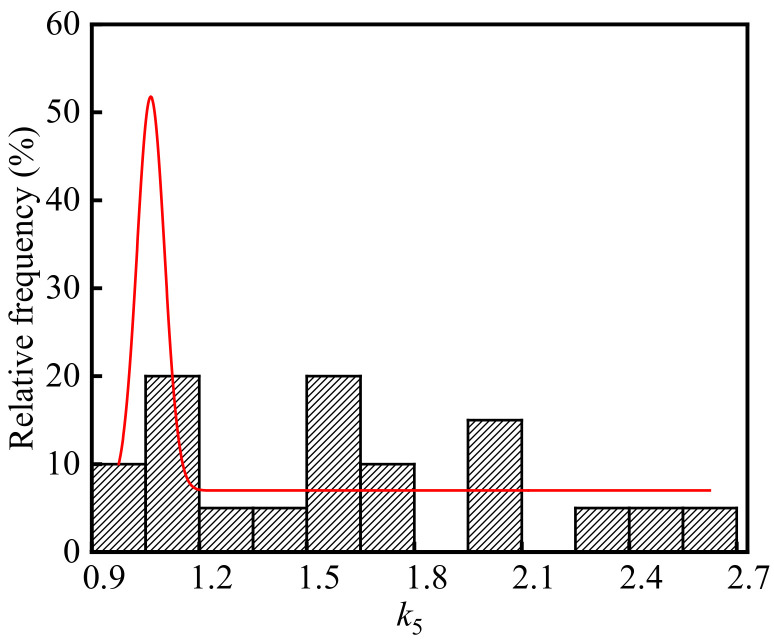
Frequency distribution histogram of *k*_5_.

Substituting the test values of the temperature, relative humidity, CO_2_ concentration, concrete compressive strength, neutralization ages in the ingot casting bay into Equation (6), the average of carbonation depth of concrete in the ingot casting bay is 9.97 mm. Combining the average carbonation depth and the neutralization depths of concrete in the ingot casting bay, *k*_5_ is calculated and the frequency distribution histogram of *k*_5_ is shown in Figure 10. As shown in the figure, the average, standard deviation, and coefficient of variation of *k*_5_ were 1.64, 0.50, and 0.31, respectively. The *p*-value of *k*_5_ was 0.30 at the significant level α = 0.05 by using Shapiro-Wilk. Therefore, *k*_5_ in the steelworks followed the normal distribution.

In conclusion, the neutralization depth of concrete in the steelworks is expressed as:

(8) $$X\left(t\right)=k_{0}k_{5}\sqrt[{4}]{T}\left(1-RH\right)RH\sqrt{C_{C}}\left(\frac{57.94}{f_{\mathrm{cuk}}}-0.76\right)\sqrt{t}$$

when *C*_S_ = 0, *k*_5_ = 1.

### 4.2. Neutralization Life Prediction of Concrete Structure

CO_2_ and SO_2_ diffuse into concrete and react with the alkaline hydration products. Once the concrete cover is completely neutralized, the protective effect on the rebars is lost, and therefore, the rebars begin to corrode. The neutralization service life of concrete takes the initial time of steel bar corrosion as the end of the structural life. Therefore, the beginning of the steel bar corrosion is the sign of neutralization service life assessment criteria of concrete structure.

(9) $$\Omega =\left\{c-x_{0}-X\left(t\right)\geq 0\right\}$$

where, Ω is the neutralization service life criteria of concrete; *c* is the thickness of concrete cover, mm; *x*_0_ is the neutralization remain, mm; *X*(*t*) is the neutralization depth of concrete, mm; and *t* is the neutralization age, d.

Therefore, the limit state function of the neutralization service life of concrete is:

(10) $$c-x_{0}-X\left(t\right)=0$$

According to the literature [43], the neutralization remain of concrete is:

(11) $$x_{0}=4.86\left(-RH^{2}+1.5RH-0.45\right)\left(c-5\right)\left(\ln f_{\mathrm{cuk}}-2.3\right)$$

**Figure 11:**
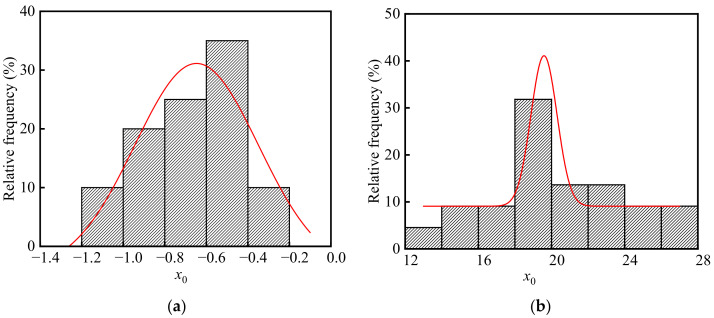
Frequency distribution histograms of *x*_0_ in the steelworks: (**a**) Ingot casting bay; (**b**) Billet bay.

Neutralization remains of concrete in the ingot casting bay and billet bay of the steelworks are calculated according to Equation (11). The frequency distribution histograms of *x*_0_ are shown in Figure 11. As shown in the figure, there were significant differences in *x*_0_ between the ingot casting bay and billet bay, and *x*_0_ in the billet bay was much larger than that in the ingot casting bay. The average, standard deviation, and coefficient of variation of *x*_0_ in the ingot casting bay were −0.67, 0.22, and −0.33 mm, respectively. As well, the average, standard deviation, and coefficient of variation of *x*_0_ in the billet bay were 20.10, 3.66, and 0.18 mm, respectively. This was because the relative humidity in the billet bay was much larger than that in the ingot casting bay. The p values of *x*_0_ in the ingot casting bay and billet bay were 0.63 and 0.89 at the significant level α = 0.05 by using Shapiro-Wilk, respectively. Therefore, both neutralization remains of concrete in the ingot casting bay and billet bay followed the normal distribution.

Substituting the calculation results of Equations (8) and (11) into Equation (10), the neutralization service life of concrete in the ingot casting bay and billet bay of the steelworks were 235.68 a and 243.07 a, respectively. Therefore, the remaining neutralization service life in the ingot casting bay and billet bay were 194.68 a and 202.07 a, respectively.

## 5. Conclusions

In this study, the environmental characteristics and corrosion degree of concrete in a 41-year-old steelworks were analyzed, and the neutralization life prediction of the concrete structure was carried out. The average temperature, relative humidity, CO_2_ concentration, and SO_2_ concentration in the steelworks were 1.32, 0.62, 1.28, and 13.93 times higher than those of the general atmospheric environment, respectively. Besides, those environmental parameters in various sections were significantly different. The temperature, relative humidity, and CO_2_ concentration in the ingot casting bay were 1.11, 0.55, and 1.23 times higher than those in the billet bay, respectively. The SO_2_ concentration in the ingot casting bay was 1.03 mg/m^3^, and there was no obvious SO_2_ in the billet bay. The appearance change of concrete in the ingot casting bay was more serious than that of concrete in the billet bay. Both the compressive strength of concrete in the ingot casting bay and billet bay of the steelworks decreased, and the compressive strength in the ingot casting bay was 1.23 times larger than that in the billet bay. The neutralization depth in the ingot casting bay was 2.35 times higher than that in the billet bay. The prediction model of the neutralization depth of concrete was established, and the remaining neutralization service life of concrete in the ingot casting bay and billet bay were 194.68 a and 202.07 a, respectively.

## Data Availability

Data are real and valid in the article.

## Supplementary Materials

The following supporting information can be downloaded at: https://www.mdpi.com/article/10.3390/ma15175893/s1, Table S1: The cover thickness, neutralization depth and compressive strength of concrete in the steelworks.
