# Supplementary material for: Corrosion Damage and Life Prediction of Concrete Structure in a 41-Year-Old Steelworks
Source: Materials (Basel). 2022 Aug 26;15(17):5893. doi: 10.3390/ma15175893 (PMC9456625; doi:10.3390/ma15175893)
Supplement: Supplementary file 1 [file materials-15-05893-s001.zip › materials-1805582-supplementary.pdf]

**Supplements**

**Table S1.** The cover thickness, neutralization depth and compressive strength of concrete in the steelworks.

| Sections | Serial number | Cover thickness /mm | Neutralization depth /mm | Rebound value of strength /MPa | Equivalent value of strength /MPa |
|---|---|---|---|---|---|
| Ingot casting bay | D1-1 | 23.7 | 25.1 | 42.4 | 28.0 |
| | D1-2 | 28.9 | 17.07 | 46.2 | 33.3 |
| | D5-1 | 46.1 | 16.41 | 52.6 | 43.3 |
| | D5-2 | 39.5 | 26.37 | 45.5 | 32.4 |
| | D11-1 | 53.5 | 15.74 | 46.2 | 33.3 |
| | D11-2 | 33.6 | 20.13 | 44.0 | 30.2 |
| | D12-1 | 36.0 | 23.36 | 50.0 | 39.1 |
| | D12-2 | 27.3 | 10.27 | 49.4 | 38.2 |
| | D15-1 | 25.3 | 16.33 | 48.7 | 37.1 |
| | D15-2 | 38.2 | 14.41 | 44.7 | 31.2 |
| | D18-1 | 40.3 | 11.27 | 45.1 | 31.8 |
| | D18-2 | 34.1 | 11.88 | 57.0 | 50.8 |
| | E3-1 | 35.3 | 9.29 | 51.4 | 41.3 |
| | E3-2 | 37.6 | 19.94 | 42.9 | 28.8 |
| | E4-1 | 18.1 | 20.92 | 48.0 | 36.0 |
| | E4-2 | 26.4 | 13.27 | 42.6 | 28.3 |
| | E11-1 | 19.3 | 15.71 | 45.6 | 32.5 |
| | E11-2 | 24.6 | 11.13 | 47.2 | 34.8 |
| | E15-1 | 22.6 | 17.15 | 47.9 | 35.9 |
| | E15-2 | 36.1 | 10.73 | 51.2 | 41.0 |
| Billet bay | 130J-1 | 33.3 | 3.36 | 43.4 | 36.6 |
| | 130J-2 | 32.3 | 11.7 | 33.9 | 18.3 |
| | 131J-1 | 30.2 | 5.43 | 48.3 | 37.9 |
| | 131J-2 | 36.7 | 5.33 | 45.3 | 33.6 |
| | 132J-1 | 35.1 | 3.04 | 43.7 | 37.6 |
| | 132J-2 | 30.3 | 8.22 | 38.5 | 23.1 |
| | 134J-1 | 38.0 | 6.93 | 44.5 | 30.9 |
| | 134J-2 | 32.4 | 9.98 | 34.2 | 18.4 |
| | 135J-1 | 31.1 | 2.49 | 34.6 | 25.4 |
| | 135J-2 | 29.1 | 10.54 | 36.0 | 20.2 |
| | 136J-1 | 42.3 | 4.88 | 25.0 | 11.8 |
| | 136J-2 | 39.1 | 9.64 | 47.3 | 35.0 |
| | 138J-1 | 30.1 | 3.64 | 46.9 | 42.1 |
| | 138J-2 | 32.6 | 10.05 | 31.3 | 15.8 |
| | 139J-1 | 36.8 | 7.61 | 51.9 | 42.1 |
| | 139J-2 | 40.8 | 10.13 | 36.9 | 21.2 |
| | 140J-1 | 26.5 | 7.35 | 44.5 | 30.9 |
| | 140J-2 | 22.5 | 8.82 | 43.8 | 29.9 |
| | 142J-1 | 32.1 | 4.75 | 39.0 | 26.7 |
| | 142J-2 | 30.4 | 5.28 | 36.1 | 21.9 |
| | 144J-1 | 24.8 | 3.64 | 48.6 | 45.2 |
| | 144J-2 | 28.6 | 9.69 | 44.2 | 30.5 |
